# Saliva Extractor

**Published:** 1877-04

**Authors:** 


### SALIVA EXTRACTOR.

To answer numerous enquiries, in reference to this appliance, devised and manufactured by the Buffalo Dental Manufacturing Co., we have only to say that it more than answers our expectations, performs its work well, and is very satisfactory wherever it has been used.

